# “Meme-ing” Across Cultures: Understanding How Non-EU International Students in the UK Use Internet Memes for Cultural Adaptation and Identity

**DOI:** 10.3390/bs15050693

**Published:** 2025-05-17

**Authors:** Yurou Zhang, Shichao Zhao, Kamarin Merritt

**Affiliations:** 1 School of Design and Creative Arts, Loughborough University, Loughborough LE11 3TU, UK; y.zhang2@lboro.ac.uk; 2 College of Business, Oregon State University, Corvallis, OR 97331, USA; kamarin.merritt@oregonstate.edu

**Keywords:** internet memes, cross-cultural adaption, cultural identity, experience-centred design

## Abstract

Non-EU international students encounter considerable challenges in social integration, cultural adaptation, and emotional well-being within UK higher education. Despite this, the role of internet memes as a form of participatory digital media in mediating these experiences has not been extensively studied. This paper examines how non-EU students at a British university utilise memes to manage cross-cultural identity and daily stressors. Employing an Experience-Centred Design (ECD) approach, our qualitative research involved 20 participants through digital cultural probes, semi-structured interviews, and co-design workshop. We discovered that memes serve a dual role: they provide emotional bridges that foster a sense of belonging through shared humour, yet they also risk exclusion due to cultural opacity. We introduce the concept of “negotiated humour”, which requires cross-cultural explanation and reduces comedic spontaneity but enhances intercultural understanding. Furthermore, we identify a continuum of meme usage that reflects different phases of acculturation, ranging from expressing frustrations to creating hybrid cultural expressions. This study contributes to cross-cultural adaptation theory by highlighting memes as boundary objects in identity negotiation. We suggest design implications for culturally sensitive platforms, such as contextual footnotes, and institutional interventions like meme-based orientation activities to exploit humour’s potential for fostering inclusive dialogue. Our research highlights how transient digital humour can provide deep insights into identity, community, and the complex dynamics of cross-cultural adaptation.

## 1. Introduction

International student populations in the United Kingdom have expanded considerably, with non-EU students now constituting a significant proportion of overseas enrolments (House of Commons Library, 2023). While these students enrich academic environments with diverse cultural perspectives, they frequently encounter challenges in adapting to unfamiliar pedagogical norms, social practices, and linguistic expectations (Smith & Khawaja, 2011; Ward et al., 2001). Such challenges, compounded by visa constraints and cultural dissonance (Halterbeck & Conlon, 2021), underscore the need to investigate how non-EU students leverage informal digital practices to navigate daily pressures, particularly through participatory media like internet memes.

This study addresses these gaps by examining how non-EU students in the UK creatively employ memes to negotiate cultural identity and belonging. Specifically, we explore how memes, particularly their visual and non-textual components, are used to express and navigate cross-cultural experiences (RQ1). We investigate the contextual and design factors influencing meme-sharing practices among non-EU students and their peers, analysing how these factors may either bridge or exacerbate intercultural divides (RQ2). Finally, we interrogate the implications of these meme-centric practices for cultural identity formation, proposing actionable strategies for institutions and platform designers to support inclusive meme use (RQ3).

Grounded in an ECD perspective (Wright & McCarthy, 2008), our methodology combines digital cultural probes, semi-structured interviews, and co-design workshop to capture the lived experiences of 20 non-EU students. This approach reveals how participants appropriate memes not only to express emotional responses to cultural adaptation, but also to forge micro-communities grounded in shared cultural references. Drawing on our own intercultural academic experiences, we approach this topic not only as researchers but also as individuals who have personally navigated cultural transitions. This positionality informs our attention to the emotional and communicative nuances embedded in meme practices. Through this lens, we introduce the concept of “negotiated humour”, which refers to instances where explaining jokes erodes their spontaneity yet sparks intercultural learning, and we also trace how meme practices mirror broader acculturation trajectories.

## 2. Related Work

### 2.1. Memes in Human–Computer Interaction

The study of internet memes within human–computer interaction (HCI) has evolved from a niche interest in viral phenomena to a critical lens for understanding participatory digital culture and its socio-technical implications. Rooted in Dawkins’ (1976) foundational concept of memes as “units of cultural transmission”, contemporary scholarship repositions memes as multimodal, user-driven artefacts that combine text, images, and videos to propagate ideas through adaptation and shared cultural resonance (Shifman, 2014). The participatory nature of internet memes aligns with Jenkins’ (2006) theory of convergence culture, where users fluidly shift between roles as consumers, adapters, and creators. This enables memes to serve as dynamic tools for identity negotiation, emotional expression, and cross-cultural dialogue. However, the ethical and cultural complexities of meme ecosystems, particularly their dual role as bridges and barriers in intercultural communication, remain underexplored in HCI literature.

The proliferation of memes is deeply intertwined with algorithmic infrastructures that prioritise engagement over cultural sensitivity. Platforms like TikTok and Instagram employ machine learning-driven recommendation systems to curate content tailored to users’ preferences, cultural backgrounds, and emotional states (Ahmed et al., 2016; Noble, 2018). While these systems enhance virality, they risk homogenising cultural expression by amplifying dominant narratives. For instance, the “Distracted Boyfriend” meme template, while globally recognisable, often loses its original satirical intent when adapted across cultures. This reflects how algorithmic prioritisation of universal formats can dilute contextual meaning (Milner, 2016). Similarly, sentiment analysis tools designed to align meme content with users’ emotional profiles may inadvertently reinforce stereotypes. A well-documented example is the “Asian Maths Genius” trope, which reduces complex identities to algorithmic caricatures, perpetuating racialised narratives (Nakamura et al., 2021; Noble, 2018).

Cross-language and multimodal processing technologies further complicate meme accessibility. Natural language processing (NLP) frameworks enable adaptive translation systems to localise memes across linguistic boundaries, yet they often fail to preserve cultural nuances. For example, automated captioning tools may misinterpret idiomatic phrases or culturally specific humour, leading to unintended exclusions (Pavalanathan & Eisenstein, 2016). Visual recognition algorithms, while effective in categorising memes based on aesthetic attributes, struggle to decode symbolic gestures or contextual references tied to specific cultural practices. This limitation is evident in platforms where gestures like the “thumbs-up” emoji, innocuous in Western contexts, are misinterpreted as offensive in regions like the Middle East (E. T. Hall, 1976). Such failures highlight the need for inclusive datasets that reflect diverse cultural lexicons and participatory design methods to mitigate algorithmic bias (Noble, 2018).

Two dominant frameworks guide meme platform design in HCI: Culturally Sensitive Design (CSD) and Participatory Design (PD). CSD, as articulated by Sun (2012), advocates integrating region-specific aesthetics, multilingual support, and contextual appropriateness into interactive systems. Platforms like TikTok have adopted CSD principles through features such as localised templates for festivals (e.g., Diwali or Lunar New Year), which enhance cultural identity while improving accessibility. However, CSD implementations risk oversimplification when culturally specific symbols, such as colours or gestures, are reduced to stereotypes. For example, the use of red and gold in Chinese New Year memes, while culturally resonant, may inadvertently exoticise traditions if divorced from their socio-historical context (Sun, 2012). PD approaches address these limitations by positioning users as co-creators. Platforms like Reddit and collaborative tools like Canva empower users to remix templates and share cultural narratives, fostering a sense of ownership (Binns et al., 2023). PD’s strength lies in its ability to surface grassroots cultural knowledge, such as how minority communities adapt memes to critique systemic inequalities. Yet, it faces challenges in equitable participation. Dominant cultural groups often dictate design priorities, marginalising underrepresented voices. Bratteteig et al. (2016) caution that without explicit safeguards, PD can replicate existing power imbalances, as seen in platforms where Western-centric humour dominates global meme exchanges.

Meme platforms grapple with systemic biases rooted in algorithmic design and data practices. Training datasets skewed toward dominant languages and cultures marginalise non-Western expressions, as evidenced by the underrepresentation of African or South Asian meme formats in mainstream platforms (Noble, 2018). Algorithmic amplification of polarising content further exacerbates exclusion. For instance, memes mocking non-native accents or regional dialects often gain traction due to their shock value, alienating users who perceive them as microaggressions (Gillespie, 2018). The dynamic evolution of meme culture complicates ethical oversight. Memes morph rapidly as users remix templates, making it difficult to predict their cultural implications. A notable example is the “OK hand gesture”, originally benign in Western contexts but co-opted as a hate symbol by extremist groups, a transformation that platforms struggled to mitigate due to delayed content moderation (Milner, 2016). This underscores the need for adaptive frameworks that balance creative spontaneity with cultural responsibility.

Despite advancements, critical gaps persist in HCI’s understanding of memes as tools for cultural adaptation. First, while studies emphasise memes’ role in political activism or viral trends (Shifman, 2014), their everyday use in transient communities, such as international students or migrant populations, remains underexplored. These groups often employ memes as coping mechanisms for cultural dissonance, yet their practices are absent from mainstream discourse. Second, the interplay between algorithmic infrastructures and micro-level identity negotiations is poorly understood. How do recommendation systems shape the visibility of culturally hybrid memes? Can participatory tools disrupt algorithmic bias, or do they inadvertently reinforce it? Finally, while ethical guidelines for meme design are emerging (Sun, 2012), few studies translate these principles into actionable interventions, such as lightweight annotations or user-driven content moderation, that preserve humour’s spontaneity while fostering inclusivity.

In conclusion, memes in HCI occupy a paradoxical space: they are both democratising forces that empower cultural expression and vectors of exclusion shaped by algorithmic and design biases. Existing research illuminates their technical foundations and social functions but falls short in addressing the lived experiences of marginalised users navigating cross-cultural adaptation. By centring cultural sensitivity, ethical accountability, and participatory equity, future work can reimagine memes not merely as digital ephemera but as vital instruments of global dialogue.

### 2.2. Cross-Cultural Theory

Cross-cultural adaptation constitutes a multidimensional process shaped by psychological, social, and institutional dynamics. At its core, this process involves navigating cultural differences, negotiating identity boundaries, and reconciling conflicting norms (Zhao, 2023). These challenges are acutely experienced by non-EU international students in the UK, who must balance cultural preservation with adaptation to unfamiliar academic and social environments. To unpack these complexities, this section synthesises foundational theories of cross-cultural adaptation, critiques their limitations in digitally mediated contexts, and positions internet memes as emergent tools for bridging cultural divides.

Berry’s (1997) acculturation model provides a seminal framework for analysing individual responses to cultural transitions. The model categorises adaptation strategies into four types: assimilation (adopting host culture norms while discarding home culture), integration (balancing both cultures), separation (rejecting host culture), and marginalisation (disengaging from both). For non-EU students, integration is often idealised as a harmonious balance. While we use the terms “home” and “host” cultures for analytic clarity, we acknowledge these categories are not fixed but continually renegotiated in practice, especially within digital spaces where hybrid references and ambiguous belonging are common (Zhao, 2020). However, practical constraints such as visa pressures, linguistic barriers, and social isolation frequently push students toward separation or marginalisation (Smith & Khawaja, 2011; Krsmanovic, 2020). For instance, Chinese students may form insular social groups using platforms like WeChat, prioritising cultural preservation over engagement with British peers (Zhang, 2022). While Berry’s model clarifies strategic choices, critics argue it oversimplifies adaptation as a linear process, neglecting the fluidity of identity in hybrid digital-physical environments (Taras et al., 2021). Hofstede’s (2001) concept of cultural distance further contextualises these challenges. His cultural dimensions, such as individualism versus collectivism or power distance, explain why students from high-context cultures (e.g., China, Saudi Arabia) may struggle with the UK’s direct communication styles or emphasis on classroom debate (E. T. Hall, 1976; Ward et al., 2001). The greater the cultural distance, the more pronounced the adaptation hurdles become. This is evident in non-EU students’ difficulties interpreting British humour or institutional norms (Halterbeck & Conlon, 2021). However, Hofstede’s framework risks reducing culture to static categories, overlooking how digital interactions enable students to reinterpret and hybridise cultural references. For example, memes blending British sarcasm with Asian visual tropes challenge rigid cultural binaries, illustrating the dynamic nature of adaptation (Shifman, 2014). Ting-Toomey’s (2005) face negotiation theory complements these perspectives by examining how individuals manage self-image (“face”) in cross-cultural interactions. In formal settings, non-EU students may adopt face-saving strategies, such as avoiding contentious debates to maintain harmony. In informal digital spaces, however, memes offer alternative avenues for face negotiation. A student might share a self-deprecating meme, using humour to acknowledge cultural misunderstanding while preserving dignity, which aligns with S. Hall’s (1990) notion of identity as fluid and performative. Memes become a stage where students simultaneously uphold and renegotiate cultural identities. This aligns with Milner’s (2016) analysis of how meme-based humour, even when misunderstood, can catalyse intercultural learning by prompting dialogue about cultural nuances—a phenomenon resonant with Ting-Toomey’s (2005) concept of face negotiation.

Theories of cultural identity construction deepen this analysis by shifting attention from fixed cultural strategies to dynamic, negotiated processes. S. Hall (1990) conceptualises identity as not stable or singular, but continually reconstituted through lived experience. This view is especially salient for non-EU students who straddle multiple cultural contexts, negotiating their sense of self across linguistic, behavioural, and social norms. Bhabha’s (1994) concept of the “third space” extends this perspective by framing identity as a hybrid formation that emerges in moments of cultural encounter and translation. For example, one participant shared a meme juxtaposing Diwali celebration with British pub culture, an act of cultural synthesis that performs bicultural belonging while disrupting binary notions of home and host cultures.

Building on these foundations, dialogical identity theory (Hermans, 2001; Hammack, 2008) offers a framework for understanding the self as composed of multiple “I-positions” that engage in internal and interpersonal dialogues. Complementing this, hybrid identity frameworks (Gamsakhurdia, 2019; Español et al., 2021) emphasise the intersubjective nature of identity work, especially in digital and post-migratory spaces. These perspectives help explain how memes operate as boundary objects through which migrant youth co-construct meaning, emotion, and cultural knowledge. Rather than simply reflecting cultural identity, memes become sites where identity is actively formed, tested, and made visible to others. Recent developments in cultural-historical psychology further enhance this theoretical articulation. Macías-Gómez-Estern (2020, 2021) introduces the concepts of “Hybrid Psychological Agents” and “Hybrid Contexts of Activity”, highlighting identity construction as an active, culturally mediated process. From this perspective, non-EU international students can be seen as hybrid psychological agents who actively negotiate meaning through memes within hybrid digital and academic contexts. This approach emphasises students’ agency in boundary-crossing practices and situated transformation, providing richer insights into their dynamic identity negotiations than traditional acculturation models alone.

Digital platforms complicate and enrich these theoretical paradigms. Traditional frameworks like Berry’s or Hofstede’s, rooted in pre-digital ethnography, underestimate the role of internet memes in mediating cross-cultural adaptation. Memes operate as cultural boundary objects (Star & Griesemer, 1989), artefacts flexible enough to hold shared meaning across diverse groups. A viral meme about UK train strikes, for example, might resonate with international students through its universal critique of bureaucracy. Simultaneously, its local references (e.g., “Northern Rail delays”) invite explanations that deepen intercultural understanding (Abidin & Zeng, 2021). This dual role highlights memes’ ambivalent power. While they democratise cultural critique through humour, they also risk alienating those lacking niche knowledge, reinforcing digital divides (Leong et al., 2010).

Critically, the participatory nature of meme culture aligns with CSD principles, which advocate for technologies reflecting users’ evolving cultural contexts (Sun, 2012). CSD moves beyond superficial localisation, such as translating text, to address deeper cultural values like collective versus individual expression. For non-EU students, meme platforms designed with CSD principles could feature adaptive templates allowing users to layer cultural symbols, such as a Chinese dragon over a British flag. They might also include embedded footnotes explaining context-specific jokes. Such innovations would balance spontaneity with inclusivity, preserving humour’s organic appeal in line with the principles of ECD (Wright & McCarthy, 2008). Algorithmic biases, however, pose risks. Platforms prioritising dominant cultural narratives may marginalise minority perspectives, as shown in Noble’s (2018) analysis of search engine biases. Participatory design methods, such as co-creating meme libraries with students, could counteract this by ensuring diverse cultural inputs shape platform algorithms.

In conclusion, cross-cultural theories provide vital lenses for analysing non-EU students’ adaptation struggles. However, their limitations in digital contexts necessitate new frameworks. Memes, as boundary objects and tools of negotiated humour, exemplify how digital artefacts mediate identity and cultural learning. By integrating CSD principles, universities and designers can harness memes’ potential to foster belonging, transforming them from trivial amusements into catalysts for intercultural dialogue.

## 3. Materials and Methods

This study adopts a qualitative, ECD approach (Wright & McCarthy, 2008) to capture the lived experiences of non-EU international students as they navigate everyday meme sharing in a British university context. ECD provides a theoretical and methodological lens for examining human experiences, emotions, and values in technology use. It is particularly suited for uncovering the informal and often fleeting nature of meme-related practices, emphasising the subjective and relational dimensions of cultural adaptation. This section outlines our research approach, participant recruitment, data collection methods, and analysis procedures, detailing how we ensured academic rigour and cultural sensitivity throughout the project. Building upon a sociocultural perspective, our study is grounded in two intersecting aims. (1) Depth of understanding: We sought to capture the nuanced interplay between humour, cultural identity, and digital communication among non-EU students. Rather than relying exclusively on interviews or surveys, we integrated multiple qualitative methods including digital cultural probes, semi-structured interviews, and co-design workshop to explore participants’ day-to-day emotional states, cultural reflections, and design aspirations. (2) Iterative and reflexive process: Consistent with ECD principles, we positioned participants as co-interpreters of their own experiences. By encouraging them to share diaries, images, and reflect on their meme use, we aspired to illuminate how memes mediate intangible elements of cross-cultural adaptation, such as negotiated humour or feelings of exclusion. In so doing, we move beyond surface-level documentation (e.g., students post memes online) to a contextual and participatory understanding of how digital artefacts figure into students’ social and emotional landscapes. By structuring the study in two complementary phases: cultural probes and interviews (Phase 1) with 12 non-EU students, followed by a co-design workshop (Phase 2) with 8 participants, this methodology offers both a granular view of meme-centric adaptation strategies and a platform for generating design solutions. The ECD perspective ensures that each participant’s subjective narrative informs the research findings, thus aligning the ensuing analysis with the real, lived complexities of cross-cultural meme sharing. The following section presents the key themes that emerged, highlighting the emotional and cultural resonances underlying everyday meme usage and the collaborative visions for more inclusive digital practices.

### 3.1. Participants and Recruitment

We recruited two cohorts. The first cohort (Phase 1) consisted of 12 non-EU international students, aged 19 to 31, each enrolled at a British university. Their national backgrounds cover a range of linguistic and cultural zones. Academic levels spanned undergraduate, postgraduate, and doctoral study, ensuring diverse everyday routines and stressors. Participants were recruited based on the following inclusion criteria: (1) aged above 18 and capable of fully understanding and consenting to the research; (2) currently enrolled as international students at Loughborough University; (3) originating from non-EU countries. These criteria helped ensure that their experiences were relevant to our research questions. Recruitment occurred via departmental mailing lists, international student societies, using a purposive sampling strategy. All twelve signed consent forms and received an information sheet detailing study aims, confidentiality measures, and their right to withdraw at any time. The second cohort (Phase 2) comprised 8 additional non-EU students, also drawn from the same university, but distinct from the first group. They were similarly required to have resided in the UK for at least two months and have previous experience of using memes. They were recruited specifically to engage in co-design workshop. This two-cohort structure enabled us to gather richly contextualised narratives from Phase 1 and then explore design possibilities in Phase 2. A summary of participant demographics for both cohorts is presented in Table 1.

### 3.2. Phase 1

#### 3.2.1. Cultural Probes

**Figure 1:**
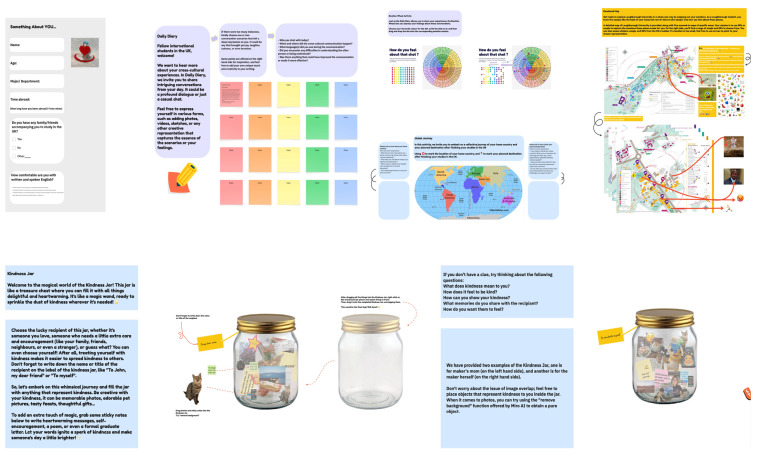
Illustration of online cultural probe via Miro.

The cultural probe method (Gaver et al., 1999) provided opportunities for the 12 Phase 1 participants to document their everyday emotional states and meme-sharing episodes. Over a two-week period, each individual used a private digital board (Miro) to post diary-like entries regarding academic routines, social activities, and personal reflections. They were prompted to embed or reference any memes encountered or shared that day, alongside short notes explaining how or why those memes resonated. Participants also utilised a colour-coded “Emotional Wheel” to express their daily moods by placing stickers or emoticons, ranging from heightened stress during exam preparation to light-heartedness upon discovering amusing local slang (see Figure 1). These open-ended tasks aligned with ECD’s focus on lived experiences and helped reveal how and when participants drew on memes for humour, self-expression, or cultural bridging. At the end of this fortnight, researchers exported the diary contents and visual attachments. The collective dataset amounted to 8–14 entries per participant, totalling over 100 textual remarks and approximately 60 images or links to memes. This materials-rich approach offered an intimate glimpse of each participant’s cultural transitions and comedic moments.

#### 3.2.2. Semi-Structured Interview

After completing the probes, the same 12 participants were invited to semi-structured interviews lasting approximately 45 to 60 min. These interviews allowed them to elaborate on or clarify their diary entries, providing narrative context for the memes they had shared. The interview guide covered issues such as the following. (1) Contexts of meme use: whether memes were sent to co-nationals, multinational groups, or domestic peers, and how humour levels varied by audience. (2) Feelings of inclusion and exclusion: instances of jokes that fell flat or led to misunderstandings, and any emotional consequences of such events. (3) Shifting meme practices: how meme usage evolved over time, for instance from an initial reliance on home-culture references to more hybrid or locally informed humour. All interviews were audio-recorded and transcribed verbatim. By comparing these transcripts with the corresponding diary data, we derived multifaceted insights into the participants’ day-to-day emotional states, the complexities of “negotiated humour”, and the role memes played in affirming or challenging cultural identity.

### 3.3. Phase 2: Co-Design Workshop

In the next phase, eight additional non-EU students (cohort 2) were recruited to participate in two identical co-design workshops, each comprising four participants to ensure manageable group dynamics, lasting approximately two hours each. The workshops began with a brief introduction, outlining the objectives and agenda to ensure participants clearly understood the purpose and expectations. Subsequently, each participant shared an item of personal cultural significance, discussing its relevance and emotional value, in order to establish mutual understanding and appreciation for each participant’s cultural background, setting a respectful tone for subsequent discussions. The main collaborative activity is scenario card creation, encouraged participants to articulate personal experiences scenarios inspired by keyword cards such as “Food”, “Weather”, “Culture”, and “Health”. Following scenario development, participants engaged in the “Meme Response” session. Here, participants either chose existing memes or created new ones to respond to the scenarios they had generated. The final activity was the “Meme Translation”, participants need to share some meme from their own cultures or personal experiences, and let others to guess the meaning. These workshops were audio-recorded, supported by researcher field notes and participants’ reflective comments. The data included sketches, digital memes, and group notes.

### 3.4. Data Analysis and Ethics

A thematic analysis (Braun & Clarke, 2006) was conducted across both phases. The research team first reviewed the diaries, interviews, and workshop transcripts individually, noting initial codes related to humour, misunderstandings, emotional coping, or local vs. home-culture references. Throughout the analysis, we maintained reflexive awareness of how our own cross-cultural perspectives might influence theme identification and interpretation, particularly in emotionally or linguistically nuanced moments. These codes were iteratively refined, merged into candidate themes, and discussed in the context of existing cross-cultural and meme literature (Berry, 1997; Shifman, 2014). Final themes, such as “negotiated humour”, “meme subcultures”, and the “acculturation curve”, consistently emerged across the data sources, albeit with differences in emphasis between the two cohorts (e.g., personal diaries vs. group-oriented design ideas). The University’s Research Ethics Committee granted approval for all procedures. Each participant received an information sheet describing the project’s aims, data handling methods, and confidentiality principles. Written informed consent was obtained, and participants retained the right to withdraw their data at any stage without penalty. All transcripts, diary entries, and digital memes were anonymised, with identifying details removed or masked. Where images included cultural or sensitive references, these were either blurred or omitted from any planned publication graphics. Emphasis was placed on fostering a respectful atmosphere, particularly during discussions of stereotypes or niche humour, so as to avoid perpetuating negative cultural tropes.

## 4. Findings

This section reports on the main themes that emerged from our two-phase data collection. We begin by outlining how we conducted the thematic analysis in practice, then present four core themes that illuminate the ways internet memes shape non-EU students’ daily experiences, emotional states, and cultural identities at a British university. Throughout, we weave in representative excerpts from diaries, interview transcripts, and workshop discussions to illustrate these themes and situate them in the broader context of cross-cultural adaptation. Following Braun and Clarke’s (2006) framework, the research team first familiarised themselves with the entire dataset, which included (a) diary entries and associated memes from the cultural probe, (b) verbatim interview transcripts, and (c) co-design workshop recordings and artefacts. We independently coded a subset of materials, noting segments that signified participants’ motivations for sharing memes, emotional responses to memes, perceived cultural misunderstandings, or references to identity formation. Through a process of axial coding, we identified links among similar codes, such as “feeling left out”, “inside jokes”, and “cultural bridging”. We consolidated related codes into candidate themes, which we tested against the data for coherence and depth of evidence. Four final themes were generated: (1) emotional self-expression and coping; (2) negotiated humour: explaining jokes, gaining understanding; (3) meme subcultures and the insider–outsider divide; and (4) adapting over time: the acculturation curve of meme practices. Although each theme stands alone, they collectively underscore the ambivalent role that memes play in supporting or complicating cross-cultural adaptation.

### 4.1. Emotional Expression and Coping

Many participants viewed memes as flexible tools for venting academic and personal emotions, especially frustrations. In P1’s diary entries, she regularly appended a crying emoji to express her mounting anxiety during her dissertation writing time. Interview data further illuminated how she and friends bonded over these comedic posts. As P1 recounted: “That period was so hectic. I’d keep putting a big cry emoji in my diary whenever I’d log how many hours I’d been writing. But also, we’d share dissertation-related memes on Instagram with friends who were rushing their own theses. The memes were mostly tongue-in-cheek about how hopeless we felt, but it made us go, ‘Oh, so we’re all in the same boat’”. These exchanges validated their shared struggles and offered a momentary reprieve from self-doubt. P2 echoed a similar function in her diary. She added a photo of railway strikes, writing: “Train strike AGAIN. My friend joked we’ll graduate before this is solved”. In her subsequent interview, the researcher should P2 a train strike related meme, P2 responded “I guess it’s silly, but if I only complained seriously, I’d feel more upset. If I can share the meme, I can at least laugh for a while, even though I am still angry”. While memes clearly offered communal coping, some participants acknowledged the risk of trivialising bigger emotional difficulties. A few admitted that the barrage of “funny meltdown memes” might deter them from seeking professional help or confiding deeper issues to mentors. Nonetheless, diary entries and interviews showed that memes remained the first place for day-to-day humour, forging a sense of “everyone’s in this together” solidarity.

### 4.2. Negotiated Humour: Explaining Jokes and Cultural Realisations

A recurring phenomenon in both the interviews and the co-design workshop was negotiated humour, referring to instances in which participants’ jokes or memes required explanation. Three illustrative examples below demonstrate how this played out in everyday and workshop settings.

#### 4.2.1. Workshop Example: Indian Participants’ Linguistic Pun

In one workshop group discussion, two Indian participants (P14 and P16, both from different Indian states) showcased a meme featuring a pun that relied on similar-sounding words in their respective Indian languages. They were amused at how the pun bridged Hindi and a southern Indian dialect (e.g., Telugu or Tamil), but the rest of the group, comprising students from China and Saudi Arabia, were perplexed. As the joke was explained, the comedic effect largely dissipated. Yet the conversation swiftly moved beyond the pun itself to the diversity of Indian languages. P14 noted: “I speak Tamil, and P16 here speaks Hindi. But I know you must think we all speak Hindi, and normally people do not ask me about the language usage in India, so they will never know!” After the explanation, the group concluded that while the comedic spark was lost in translation, the explanation uncovered a cultural reality: India is linguistically vast, meaning local puns could emerge from countless dialects. Students from other countries admitted they had assumed “Indian is just one language”, and left the session with a deeper understanding. Workshop notes showed that participants suggested a “footnote” or short label could be attached to such memes online, so novices might at least read a succinct explanation without derailing the comedic flow entirely. P15: “Although there is no explanation about some memes from other cultures, I still want to watch it, but in most situations, I don’t get it, and I hope someone can tell the story behind this meme”.

#### 4.2.2. Workshop Example: Football References

**Figure 2:**
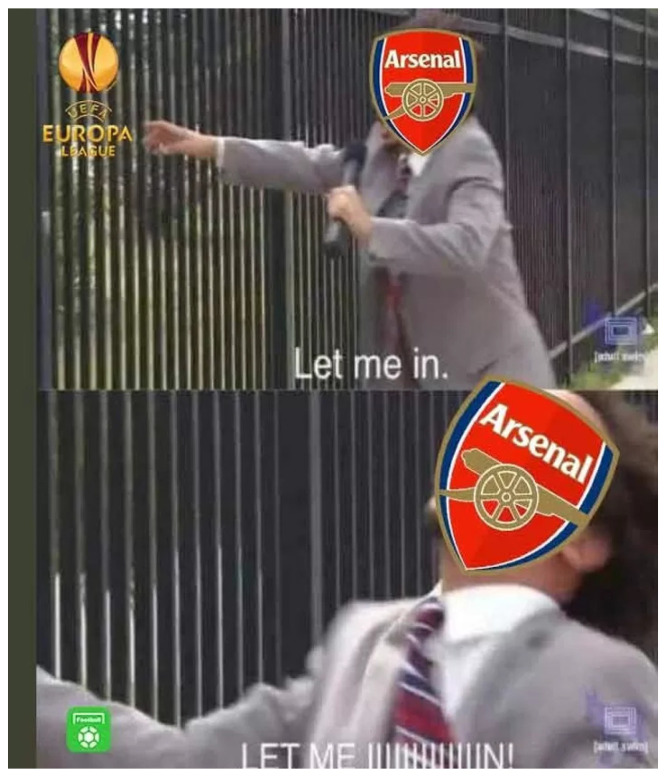
Arsenal Europa League meme shared by Participant P11.

Another workshop scenario involved a Chinese male student (P11), a long-term UK resident and avid football fan, who shared a viral Arsenal-themed meme from the 2023 Europa League season (see Figure 2). The image depicted the Arsenal crest alongside the text: “EUROPA LEAGUE: Let me in. Arsenal”. This meme satirised the club’s fraught history in European competitions, resonating deeply with participants familiar with British football banter. A Japanese peer (P19) immediately recognised the self-deprecating humour, laughing at Arsenal’s recurring struggle to advance in the tournament. However, participants unfamiliar with British football culture, particularly from Thailand and Iranian, were initially puzzled. Once explained that “Arsenal” was a London-based team often joked about in internet football memes, the comedic punch weakened, yet they began asking about how popular football was in England. The group spent several minutes discussing regional team rivalries and local chants, culminating in an observation: “Unless you follow Premier League ‘banter culture’, this meme just looks like a random complaint”. (P19). P11 later reflected: “It’s not just about the joke. It’s about the entire British football culture. People here talk about offside controversies like it’s the end of the world. Once you ‘get it’, you find the memes funnier”. This comedic breakdown thus triggered discussions about how football banter saturates British media, helping novices to appreciate broader cultural references lurking behind memes. Workshop transcripts recorded participants concluding that optional “fan footnotes” or short references to the team or chant could facilitate cross-cultural understanding.

#### 4.2.3. Workshop Example: An Arab Participant’s Gesture-Based GIF

**Figure 3:**
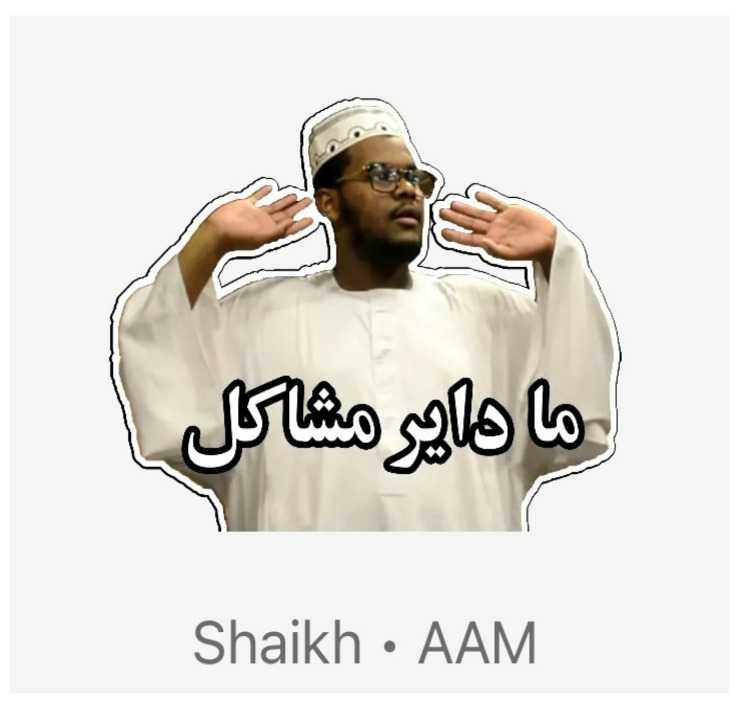
Sudanese gesture-based meme shared by P12.

In the “meme translation” activity, P12 (female, Arab) presented a caption-less GIF depicting a Sudanese individual raising both arms upwards (Figure 3). Other participants guessed it was a gesture of victory, celebration, or dancing. P12 then revealed that in her experience, this gesture actually signifies “none of my business”. According to her explanation, in Sudan, raising one’s arms like that often implies a laid-back disavowal of responsibility. She remarked: “They do this arm thing to say, ‘I’m out—I’m not getting involved’. In our culture, we think Sudanese people are so cool”. Once the group realised the meaning was essentially the opposite of “victory”, they launched into a discussion about hand signals across cultures. Participants expressed surprise at how the same physical gesture can have radically different connotations. Some used the example of the pinkie finger: P14 noted that holding up one’s pinkie in parts of India can mean “I need the toilet”, while P12 pointed out that in China, it may signal contempt. P18 added that in Japan it can represent “my partner” whereas P12 observed that in her Arab community, raising one’s pinkie may indicate a friendly invitation. These revelations resonated with the concept of cultural relativity in gestures, underscoring that comedic memes relying on a certain posture or motion can easily confuse those from other contexts. This example showcases how a seemingly straightforward GIF (without text) required an elaborate explanation, “killing” the instant laugh but illuminating a wider set of differences in bodily symbolism. As participants discovered how a single posture might mean celebration, dismissal, or romantic involvement depending on the region, they recognised that meme-based gestures are not always universal. Ultimately, they saw how comedic “misfire” is less about comedic failure and more about an entry point to intercultural discovery. The discussion ended with participants praising the workshop for “exposing how we can’t assume any universal understanding is universal”.

**Figure 4:**
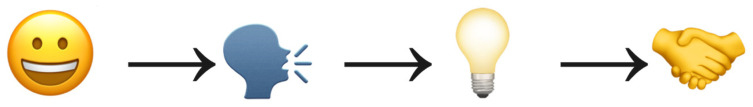
Cycle of negotiated humour in cross-cultural meme-sharing (meme-sharing → confusion → explanation → cross-cultural understanding).

Across these examples, negotiated humour emerges as a double-edged phenomenon. Explaining jokes typically “kills the spontaneity”, as P14 put it, but paves the way for deeper mutual understanding and cultural awareness. Participants consistently framed these comedic breakdowns as temporarily embarrassing but ultimately valuable, expanding group knowledge, whether it is the breadth of Indian dialects, British football fervour, or Sudanese gestures. In terms of emotional outcomes, some described an initial awkwardness (“I had to ask for a translation and felt clueless”), yet many also relished “aha” moments that drew them closer. From a design standpoint, they suggested minimal interventions, such as footnotes, disclaimers, or orientation-based meme exchanges, that might facilitate comedic bridging without over-regulating creativity or spontaneity. Hence, negotiated humour illustrates how digital memes, far from mere amusements, can spur profound intercultural dialogue. Students come to appreciate how comedic references hinge on local knowledge or assumptions, and in explaining them, they collectively build a patchwork of cultural literacy. We summarised this process in the cycle of negotiated humour (Figure 4). This process echoes Ting-Toomey’s (2005) “face negotiation” approach, in which brief “losses” of comedic face evolve into meaningful cross-cultural bonds.

### 4.3. Meme Subcultures and the Insider–Outsider Divide

A recurring theme across interviews and the co-design workshop was the way distinct meme subcultures formed among non-EU students, creating insider groups grounded in shared references. In his interview, P6 (male, Chinese, 26, PhD in Chemical Engineering) highlighted how he and several compatriots had established a WeChat group centred on a popular online game. He recounted frequently saving comedic screenshots and memes from that group, where jokes played on game mechanics and Chinese internet slang. However, P6 noted that he “would never post these in a more general chat” because others would not understand the context: “Our gaming group has specific terms, like different characters can represent one feature. But if I showed it to my lab mates who aren’t gamers, they’d just be confused”. This in-group exclusivity extended to other domains as well. Another example came from participants who regularly swapped food-related memes focusing on, for instance, stickers about authentic Chinese cuisine or Indian regional dishes, content that rarely resonated outside their cultural circle. P2 explained that she and three Chinese friends often joked about “the ways we can’t replicate our home cooking”, using memes that mock British supermarkets’ limited spice options. While these comedic posts strengthened their shared sense of nostalgia, P2 admitted her European or Middle Eastern acquaintances “would see the same meme and not even slime, because they don’t see that a big deal”.

Such examples demonstrate both the comfort and the fragmenting potential of subcultures. Students gained solace and laughter through memes referencing familiar pastimes, linguistic puns, or food traditions; yet those not privy to the references felt excluded. Discussions in the workshops similarly pinpointed this insider-outsider dynamic. Several attendees expressed how they stuck to culturally “safe” corners online, rarely venturing into cross-national meme spaces. They also acknowledged that bridging subcultural divides could be a missed chance for intercultural learning, since external viewers may never receive explanations or invitations to “join the joke”. These findings underscore how meme subcultures can safeguard cultural identity but inadvertently limit broader community integration.

### 4.4. The Acculturation Curve: Evolving Meme Practices over Time

Several participants reflected in interviews and workshops that their meme usage had shifted since they first arrived in the UK. During interviews, P4 stated that when he began his programme, he mostly shared memes with home-culture references, he maintained the habit of searching Chinese based memes. However, after a few months, he gradually introduced more hybrid memes that blended Chinese comedic elements with British campus in-jokes. Reflecting on this progression, he explained: “Early on, I was stuck in a loop of seeing everything ‘foreign’ with Chinese memes. Now I’ll say, ‘Queueing in the UK is next level’, and use an English meme format. It’s me picking up local humour styles”. Participants like P1 similarly recalled in her interview that her comedic sensibilities broadened as she interacted more with domestic students. She mentioned how she had started receiving and sending memes primarily with her Indian circle, using meme from their shared experiences. Over time, though not directly tracked in her short diary entries, she noticed a “big shift” in her comedic style once she grasped certain British slang and local cultural norms. According to P1: “After staying here about two months, I finally get some jokes from local cultures, like British grocery security stuff. And gradually I can understand some British humour”.

This self-reported evolution of meme usage points towards an acculturation curve in comedic expression. A few co-design workshop participants also echoed that, upon arriving, they stuck to memes from home-culture social media (e.g., Weibo, Indian film parodies, or Ghanaian comedic references). As they became more comfortable with local slang or pop culture, they tested out references that resonated with mixed-nationality peers. Still, not every participant shifted towards hybrid humour. P9 told us in his interview that he mostly retained a “China-centric meme repertoire”, continuing to share content from WeChat circles without integrating local British or European comedic elements. He felt those local references “weren’t worth the effort” to learn, especially given his short stay and heavy coursework.

While our diaries alone did not capture a long arc of changing meme usage, participants’ own recollections in interviews and workshop reflections suggested that comedic practices often evolve as students become more acclimated. Some eventually found themselves able to adopt or remix local memes, bridging both cultures in their humour. Others largely maintained their original comedic enclaves. This idea of a “meme-based adaptation curve” parallels broader cultural integration processes described by Berry (1997), though manifested in ephemeral digital exchanges rather than formal cultural milestones.

## 5. Discussion

This study illuminates how non-EU international students use memes not merely as short bursts of comedic relief but as symbolic conduits for negotiating cultural belonging, emotional identity, and social boundaries. While Section 4.2, Section 4.3 and Section 4.4 established the core phenomena including emotional venting, negotiated humour, subcultural enclaves, and an evolving comedic repertoire, this discussion section moves beyond restating findings to synthesise them with cross-cultural adaptation theories (Berry, 1997; Ward et al., 2001) and digital media perspectives (Jenkins, 2006; Shifman, 2014), highlighting the often-ambivalent power of memes to both unify and fragment campus communities.

### 5.1. Theoretical Contributions and Interpretations

A central theme that arises from our data is the inherently ambivalent function of memes as cultural boundary objects. Although prior work often underscores their ability to unify audiences (Shifman, 2014), our findings suggest that memes can simultaneously foster inclusion and exclusion, act as a conduit for negotiated humour, and occasionally perpetuate stereotypes. Berry’s (1997) acculturation model helps clarify how memes support students who oscillate between the desire to integrate local comedic elements and the need to maintain home-culture references. One participant’s reflections on blending British soap-opera imagery with jokes from their home country illustrate the creation of a “third space” (Bhabha, 1994), wherein cultural norms are reframed rather than simply replaced. This hybridity underscores how memes can become performative artefacts of bicultural identity, rather than mere pieces of online ephemera.

Building on these theoretical foundations, we further draw on recent developments in cultural-historical psychology to deepen our understanding of identity work in digital, migratory contexts. Dialogical identity theory (Hermans, 2001; Hammack, 2008) conceptualises the self as composed of multiple, shifting “I-positions” that interact through internal and interpersonal dialogues. Among our participants, meme sharing often exemplified this dialogicity, for instance, using humour in a heritage language while also recontextualising it for cross-cultural peers. Similarly, hybrid identity frameworks (Gamsakhurdia, 2019; Español et al., 2021) emphasise the performative and emergent nature of identity, particularly in “third spaces” where cultural categories blur. Our findings show how students use memes to enact such hybridity, blending British visual tropes with culturally specific meanings to produce content that resists fixed categorisation. To further extend this perspective, we incorporate Macías-Gómez-Estern’s (2020, 2021) concepts of hybrid psychological agents and hybrid contexts of activity, which foreground individuals’ agency in co-constructing identity across overlapping cultural systems. These frameworks help explain not only how participants inhabit hybrid identities, but also how they actively mediate meaning through digital practices. For instance, the phenomenon of negotiated humour illustrates this agency vividly: rather than experiencing cross-cultural misunderstandings as mere comedic failure, students often used them as opportunities for reflection, explanation, and shared learning. In this way, humour functions as a boundary-crossing practice that fosters intercultural empathy and what Macías-Gómez-Estern describes as situated transformation, a dynamic reconfiguration of meaning that emerges within hybrid social and institutional contexts.

Yet hybridity alone does not capture the full emotional and communicative dynamics of meme sharing. The process of “negotiated humour” repeatedly surfaced in our data, aligning with Ting-Toomey’s (2005) face-negotiation theory. While memes often rely on immediate comedic impact, many students reported that explaining jokes to cross-cultural peers could “kill the laughter” but simultaneously deepen cultural awareness. Far from trivial, these moments of halting humour often spurred important conversations about language, social references, and historical nuance. Ironically, the initial comedic breakdown often gave way to a more collaborative form of laughter, reflecting a shared investment in mutual understanding. Such interactions echo Gudykunst’s (2005) emphasis on anxiety and uncertainty management in intercultural communication, revealing how “failed jokes” can transform into opportunities for connection and empathy.

A further theoretical layer emerges around the ways memes can create subcultures that reinforce in-group/out-group distinctions. While participants found comfort and camaraderie in smaller circles of shared comedic tastes, this practice could inadvertently exclude those unfamiliar with niche references. These meme enclaves function as intense micro-communities that reinforce a sense of insider solidarity, yet risk perpetuating parallel forms of cultural fragmentation (Milner, 2016). Certain participants also highlighted a disquieting aspect: the circulation of memes that unwittingly caricatured certain ethnic or national identities, underscoring that humour can mask or legitimise stereotyping (Phillips & Milner, 2021; Shifman, 2014). Thus, despite memes’ capacity to enliven cultural exchange, they also pose ethical challenges in an academic environment committed to inclusivity and respect. Collectively, these findings enrich scholarship on digital culture (Jenkins, 2006; Shifman, 2014) by illustrating that memes are not merely lighthearted diversions but multifaceted signifiers of cultural affiliation, emotional expression, and potential misrepresentation. They serve as loci of identity negotiation, allowing non-EU students to signal hybrid belonging while simultaneously demarcating boundaries around who “gets the joke”. Whether these dynamics lean towards bridging or fragmenting groups depends substantially on how participants navigate the comedic codes inherent in meme culture.

### 5.2. Practical Implications for Design and Support

The ambivalent roles that memes play in cross-cultural settings point to several opportunities for institutions and platform developers aiming to foster inclusive online environments. One recurring suggestion was to incorporate low-threshold design features, such as footnotes, hover-over captions, or user-generated annotations, that could clarify cultural references. These small interventions would reduce the alienation students described when encountering memes laden with local slang or unfamiliar historical allusions. While care must be taken to avoid overregulation that dampens spontaneous humour, such solutions embody a culturally sensitive design ethos (Sun, 2012), encouraging the broadest range of students to participate in meme-based exchanges. Universities could also integrate memes into orientation activities, student-led cultural festivals, or co-curricular workshops. Rather than regarding memes as frivolous, educators and student support services might leverage them to open dialogue about academic stress, social norms, and the nuances of British life. By inviting new arrivals to co-create or adapt memes that depict their initial impressions (whether confusion at local customs or lighthearted jabs at coursework), institutions can normalise the sharing of cultural challenges through accessible, low-stakes humour. Such activities have the added benefit of nudging isolated meme enclaves towards a more collaborative, cross-cultural sphere, as students discover new comedic repertoires and shared experiences.

Moreover, student mental health and well-being services could harness memes as informal “emotional barometers”, noting how they often reflect collective moods and everyday anxieties. Prompts such as “Share a meme that captures your current feelings” can serve as conversation starters in counselling sessions or peer-support groups, especially for individuals who may be hesitant to voice their struggles in a purely verbal format. This approach is not a substitute for professional interventions but can function as a low-barrier entry point for identifying and empathising with students under stress. Nonetheless, it remains vital to acknowledge that humour can also mask deeper or more serious issues; integrating meme-sharing with dedicated support channels would help prevent the trivialisation of genuine emotional distress.

An additional challenge concerns freedom of expression and cultural sensitivity. Automated moderation systems often struggle to interpret sarcasm, regional idioms, or parody, features that define meme culture, thus risking misclassification or unwarranted censorship. Community-driven moderation, shaped by a diverse range of stakeholders, may offer a more adaptive model that balances comedic spontaneity with respect for cultural boundaries. Jenkins’s (2006) vision of participatory culture aligns well with this approach, suggesting that collective norms, rather than top-down rules, can be an effective means of guiding ethical meme creation and circulation. Ultimately, thoughtful design choices and institutional initiatives can help ensure that memes enrich international students’ cultural journeys, rather than exacerbating misunderstandings or reinforcing stereotypes. Beyond design interventions, our findings suggest potential value for educational and policy settings. Institutions might incorporate meme-based storytelling or explanation activities into cross-cultural training or orientation workshops to encourage early interaction across cultural groups. Educators may draw on meme discourse as a form of culturally relevant pedagogy that acknowledges digital fluency and informal literacies. Mental health services could also use meme creation as a reflective activity in student well-being programmes, particularly for students who may struggle with verbal expression in a second language.

### 5.3. Methodological Reflections, Limitations, and Future Research

A key methodological strength of this study lies in its integration of cultural probes, semi-structured interviews, and co-design workshops to capture the highly contextual and often fleeting nature of meme-sharing among non-EU international students. The cultural probes allowed participants to record daily meme encounters and their emotional undertones, offering an immediate and situated account of digital humour practices. Semi-structured interviews enabled deeper narrative exploration, while the co-design workshops invited participants to become co-researchers, generating creative strategies for reducing cultural opacity and social exclusion (Zhao, 2022). Despite these benefits, privacy emerged as a recurring concern: several individuals hesitated to share memes from personal chats or group forums, underscoring the need for ethically sensitive methods, such as anonymised diaries, curated “meme boards”, or partial screenshots that mask personal identifiers, to safeguard participants’ sense of security. In line with these observations, participants themselves and researchers generated design-related reflections that illuminate how platforms and institutions might support a more inclusive meme culture. Many advocated “lightweight cultural footnotes” or hover-over explanations to mitigate confusion without eroding comedic spontaneity, especially for jokes referencing home-cuisine puns or local public transport gripes. Others proposed incorporating meme-sharing sessions into new-student orientation, enabling early, low-stakes opportunities to “explain the joke” and thereby reduce later embarrassment. At the university level, suggestions included “meme festivals” or themed exhibitions, opening subcultural enclaves to broader campus dialogue and fostering intercultural curiosity. However, participants also stressed that over-regulation, for instance, heavily policing which memes are “allowed” or mandating footnotes for every post, which could drive humour into more private, insular channels. Achieving a balance between spontaneity and clarity thus became a recurrent theme in these design reflections. As international researchers with lived experience navigating transnational education systems, we acknowledge that our positionality shaped every stage of this project, from the framing of research questions to the interpretation of “negotiated humour”. Our own intercultural backgrounds heightened our sensitivity to moments of embarrassment, exclusion, and humour lost in translation. At the same time, we remained reflexive about the potential biases this standpoint could introduce, particularly in interpreting participants’ intentions or framing cultural contrasts. This continuous reflexivity informed our efforts to engage ethically and critically with the cultural specificity of digital meme practices.

Despite offering a window into these creative possibilities, the study is not without limitations. Firstly, we initially utilised the broad categorisation of ‘non-EU’ students, which was largely driven by visa regulations and institutional criteria, but this binary framework does not fully capture the nuanced cultural diversity of our participants. Although ‘non-EU’ provided a convenient starting point, it inevitably obscured the significant variations in cultural backgrounds and experiences among students. Their varied meme preferences and interpretations point to a need for more nuanced approaches to cultural identity in future research. Secondly, all participants were drawn from a single British university, which may limit the generalisability of our findings to other institutions with different cultural compositions, infrastructures or academic pressures. Although our recruitment strategy captured a range of ages, academic levels and national backgrounds, the unique institutional context and local culture may have influenced the dynamics of meme sharing. Future studies could adopt a multi-site or longitudinal design, and incorporate domestic student perspectives, to better understand how meme-based communication operates across diverse cultural and institutional settings. Our data collection was also conducted over a limited time frame, capturing only a partial arc of students’ adaptation journeys. As a result, the self-reported “acculturation curves” of meme-sharing, which shift from home-centric to hybrid comedic repertoires, rely partly on retrospective accounts rather than continuous observation. Moreover, the voices of domestic UK students remained peripheral in our dataset; future research could therefore broaden the lens to examine how local peers perceive, interpret or co-create memes in multicultural campus settings. Finally, although methods such as cultural probes and emotional wheels were employed to capture participants’ affective experiences, these instruments were originally designed on the basis of Western cultural assumptions and may therefore exhibit interpretative biases in different cultural contexts. Future research should consider localising these tools or developing new, culturally sensitive instruments, and should validate their cross-cultural applicability through experimental design. The concept of “portability”, as discussed by Jung Joo Lee (2012), could be especially relevant in this regard, as it highlights the importance of adaptable and flexible research tools in diverse cultural settings. Employing a mixed-methods approach (combining qualitative and quantitative techniques) to assess the cross-cultural stability of these instruments would further enhance methodological rigour.

Building on these limitations, there is ample scope for future work. A multi-campus or longitudinal study could determine whether the design ideas proposed here, such as cultural footnotes or meme-based orientation, indeed facilitate deeper cross-cultural engagement and reduce exclusionary in-jokes. Researchers might also delve further into the ethical and political dimensions of meme circulation, particularly when humour intersects with stereotypes, microaggressions, or unequal power relations. Critical discourse analysis, combined with iterative co-design, could help reveal hidden biases encoded in seemingly innocuous memes. Additionally, given the rapid popularity of short-form video platforms like TikTok, scholars could explore whether these ephemeral, algorithmically amplified formats exacerbate or mitigate the same cultural barriers observed in static memes. By addressing these questions, future studies may sharpen our theoretical and practical understanding of how digital humour negotiates cultural identities in increasingly globalised educational contexts.

## 6. Conclusions

This research demonstrates that, for non-EU international students, internet memes go far beyond mere entertainment. They operate as everyday acts of self-expression, tools for building communal rapport, and even conduits of unplanned cultural education. The diaries and interviews revealed how students harness comedic references to express collective frustrations, such as those involving dissertations, train strikes, and local norms, while co-design workshop demonstrated how comedic collisions can lead to surprising cultural epiphanies. Although comedic spontaneity occasionally “fails” in intercultural contexts, participants widely agreed that such breakdowns often lead to deeper mutual understanding. Memes thus emerge as boundary objects (Star & Griesemer, 1989) that reveal assumptions, bridge knowledge gaps, and prompt curiosity about linguistic or gesture-based nuances. Yet this study also highlights the subcultural enclaves that thrive on insider humour, creating pockets of comfort at the expense of broader integration. While these enclaves buffer homesickness and stress, they can limit social interaction with students from different national backgrounds. The notion of an “acculturation curve” in meme usage, as reported retrospectively in interviews, further complicates assumptions about linear adaptation. Comedic references can shift from purely home-centric vantage points to more hybrid, localised forms, although not all individuals follow the same track.

In turning these findings into action, we recommend that universities and platform designers adopt modest interventions such as optional disclaimers, orientation-based meme co-creation, or user-driven context tags that can mitigate unintentional alienation without undermining humour’s spontaneity. Future inquiries might test such features in campus digital ecosystems, track meme-sharing over a longer duration, or investigate how domestic students reciprocally integrate non-EU comedic references. By spotlighting the interplay between ephemeral internet humour and profound cultural negotiation, this work encourages a revaluation of memes as essential parts of the cross-cultural student experience, worthy of both empirical study and design innovation.

## Figures and Tables

**Table 1:** Participant Demographics for Phase 1 and Phase 2 (Note: P1–P12 = Phase 1, P13–P20 = Phase 2).

Participant	Gender	Nationality	Age	Department	Academic Degree	Time in the UK (Months)
P1	Female	Indian	28	Business	Master	11
P2	Female	Chinese	31	Business	Master	10
P3	Male	Ghanaian	24	Computer Science	Master	11
P4	Male	Chinese	24	Sports	Master	12
P5	Female	Chinese	27	Design	PhD	11
P6	Male	Chinese	26	Chemical Engineering	PhD	48
P7	Male	Japanese	19	Language	Bachelor	3
P8	Male	Japanese	20	Language	Bachelor	3
P9	Male	Chinese	27	International Relations	Bachelor	4
P10	Female	Uzbekistani	22	Business	Master	36
P11	Male	Chinese	20	Architecture	Bachelor	120
P12	Female	Saudi Arabian	27	Arts	PhD	14
P13	Female	Chinese	27	Design	PhD	13
P14	Male	Indian	26	Sports	Master	10
P15	Female	Arab	32	Design	PhD	8
P16	Female	Indian	25	Arts	Master	10
P17	Female	Thai	28	Design	PhD	20
P18	Male	Chinese	24	Engineering	Bachelor	25
P19	Male	Japanese	26	International Relations	Bachelor	5
P20	Female	Iranian	31	Design	PhD	8

## Data Availability

Data are contained within the article.
